# Efficacy and safety of traditional Chinese herbal medicine combined with HAART in the treatment of HIV/AIDS: A protocol for systematic review and meta-analysis

**DOI:** 10.1097/MD.0000000000028287

**Published:** 2021-12-30

**Authors:** Zhenzhen Qian, Yujin Zhang, Xiaoli Xie, Junwen Wang

**Affiliations:** a Hunan University of Chinese Medicine, Changsha, Hunan, China; b The Second Affiliated Hospital of Hunan University of Chinese Medicine, Changsha, Hunan, China.

**Keywords:** highly active antiretroviral therapy, human immunodeficiency virus/ acquired immunodeficiency syndrome, meta-analysis, systematic review, traditional Chinese herbal medicine

## Abstract

**Background:**

Acquired immunodeficiency syndrome (AIDS) is one of the infectious diseases pandemic in the word. Traditional Chinese herbal medicine, as an alternative and complementary therapy of highly active antiretroviral therapy (HAART), has been put into the treatment of human immunodeficiency virus (HIV)/AIDS over 30 years due to its good therapeutic effects and high safety, while there is a lack of evidence-based medicine support. The purpose of this study is to explore the efficacy and safety of traditional Chinese herbal medicine combined with HAART for HIV/AIDS patients.

**Methods:**

We will search all randomized controlled trials of Chinese herbal medicine combined with HAART in the treatment of HIV/AIDS from electronic databases including PubMed, Cochrane Library, Embase, China National Knowledge Infrastructure, WanFang, China Science and Technology Journal Database and Chinese Biomedical Literature Database from inception to December 31, 2021. Literature screening will be conducted through EndNote software, and data extraction will be processed according to inclusion and exclusion criteria by two independent researchers. We will use Review Manager 5.4 and Stata 16 software for data analysis and publication bias test.

**Results:**

This systematic review and meta-analysis will provide a high-quality evidence for the efficacy and safety of traditional Chinese herbal medicine combined with HAART in the treatment of HIV/AIDS.

**Conclusion:**

The conclusion of this review will provide an objective assessment to evaluate whether Chinese herbal medicine integrated with HAART has the effect of improving the efficiency and depressing the toxicity.

**Registration number:**

INPLASY2021110082.

## 1 Introduction

Acquired immunodeficiency syndrome (AIDS), defined as a kind of chronic contagious and inflammatory disease,^[1]^ which is caused by human immunodeficiency virus (HIV) infected with decreased CD4 cell count and abnormal continuous activation of immune system as the main pathological changes.[^2^,^3^,^4^] Highly active antiretroviral therapy (HAART), acted as the mainstream therapy in the treatment of HIV/AIDS, plays a very important role in reducing HIV viral load,^[5]^ lowering morbidity and mortality of AIDS.^[6]^ However, clinical studies have found that long-term use of antiviral drugs may lead to a series of adverse reactions such as renal dysfunction,^[7]^ osteoporosis,^[8]^ neurocognitive disorder,^[9]^ even cancer,^[10]^ which can directly affect clinical efficacy and medication compliance of patients.

Traditional Chinese herbal medicine, as a supplement to HAART, has been applied into the treatment of HIV/AIDS for a long history.^[11]^ It has been showed that Chinese herbal medicine combined with HAART not only can delay the onset of HIV infection, improve clinical symptoms and signs, increase CD4 cell count, promote immune function reconstruction,^[12]^ but also can reduce the incidence of serious opportunistic infections and improve quality of life of patients with less side effects.^[13]^ However, it has not been systematically verified that traditional Chinese herbal medicine in combination with HAART can improve clinical efficacy and reduce adverse reactions. Therefore, the aim of this review is to objectively evaluate the efficacy and safety of Chinese herbal medicine combined with HAART for patients with HIV/AIDS, in order to provide reliable evidences and valuable references for clinical doctors and researchers to make better medical decisions and conduct further studies.

## 2 Methods

### 2.1 *Study* registration

This protocol of systematic review and meta-analysis has been drafted according to the preferred reporting items for systematic review and meta-analysis protocols.^[14]^ Moreover, it has been registered on the International Platform of Registered Systematic Review and Meta-Analysis Protocols website and registration number is INPLASY2021110082.

### 2.2 Inclusion and exclusion criteria

#### 2.2.1 Types of studies being included

All randomized controlled trials (RCTs) in regard of the efficacy and safety of Chinese herbal medicine combined with HAART in the treatment of HIV/AIDS will be included in this systematic review. There will be no restrictions imposed on the country of study, the status of publication and the number of population, but the language will be limited to Chinese and English.

#### 2.2.2 Participants of studies

The participants in all studies will be composed of individuals who are over 18 years old with HIV/AIDS which is diagnosed by a clinician, or using any recognized diagnostic criteria. No restrictions on gender, race, religion, and sexual behavior will be imposed.

#### 2.2.3 Interventions

The treatment group will receive the therapy of Chinese herbal medicine in combination with HAART, at the same time, the control group will only accept HAART, placebo, or no intervention. Chinese medicine includes traditional Chinese medicine (TCM) formula granule, Chinese herbal pieces and Chinese patent medicine, while HAART is consisted of three or more conventional antiviral drugs. There will be no limited on dose, frequency and duration of treatment.

#### 2.2.4 Outcomes

(1) Main outcomes. The primary outcomes were CD4+ cell, CD8+ cell, CD4+/CD8+ cell ratio, CD45RO cell, CD45RA cell and quality of life.
(2) Secondary outcomes. Secondary outcomes included Karnovskey Score, opportunistic infections (The frequency of pulmonary infection, oral candida albicans infection, intestinal infection and other opportunistic infections), and adverse effects (The frequency of vomiting, rash, abnormal liver and kidney function).

#### 2.2.5 Exclusion criteria

(1) Animal experiments, reviews, case reports, meta-analysis, duplicated studies, and non-randomized controlled trials.
(2) The data was incomplete, interventions were failed to meet requirements and outcome indicators were inconsistent.

### 2.3 Information sources

We will search the following databases from inception to December 31, 2021 for relevant English and Chinese language literature: PubMed, Cochrane Library, EMBASE, China National Knowledge Infrastructure, WanFang Database, China Science and Technology Journal Database and Chinese Biomedical Literature Database. RCTs with regard to the efficacy and safety of Chinese herbal medicine integrated with HAART in the treatment of HIV/AIDS will be searched in those databases by experienced researchers.

### 2.4 Search strategy

In order to process a comprehensive search, two expert authors will search those databases with the following search strategies: “HIV,” “HIV infections,” “human immunodeficiency virus,” “acquired immunodeficiency syndrome,” “Chinese medicine,” “Chinese drug,” “Chinese herb,” “ TCM,” “highly active antiretroviral therapy,” “HAART,” and so on. The search strategy for PubMed as an example is shown in Table 1.

**Table 1:** Search strategy in PubMed database.

#1	“HIV” or “HIV Infections” [Mesh]
#2	“hiv” or “hiv1” or “hiv2” or “human immunodeficiency virus” or “human immunedeficiency virus” or “human immuno-deficiency virus” or“human immune-deficiency virus” [Title/Abstract]
#3	**“Acquired Immunodeficiency Syndrome” [Mesh]**
#4	“acquired immunodeficiency syndrome” or “acquired immunedeficiency syndrome” or “acquired immuno-deficiency syndrome” or “acquired immune-deficiency syndrome” or “AIDS” [Title/Abstract]
#5	#1 or #2 or #3 or #4
#6	“Medicine, Chinese Traditional” or “Drugs, Chinese Herbal” or “Plant Extracts” or “Plants, Medicinal” or “Herbal Medicine” [Mesh]
#7	“TCM” or “chinese medicine” or “chinese drug” or “chinese herb” or “alternative medicine” or “complementary medicine” [Title/Abstract]
#8	#6 or #7
#9	“Antiretroviral Therapy, Highly Active” [Mesh]
#10	“highly active antiretroviral therapy” or “antiretroviral therapy” or “HAART” [Title/Abstract]
#11	#9 or #10
#12	#5 and #8 and #11

### 2.5 Literature screening

**Figure 1:**
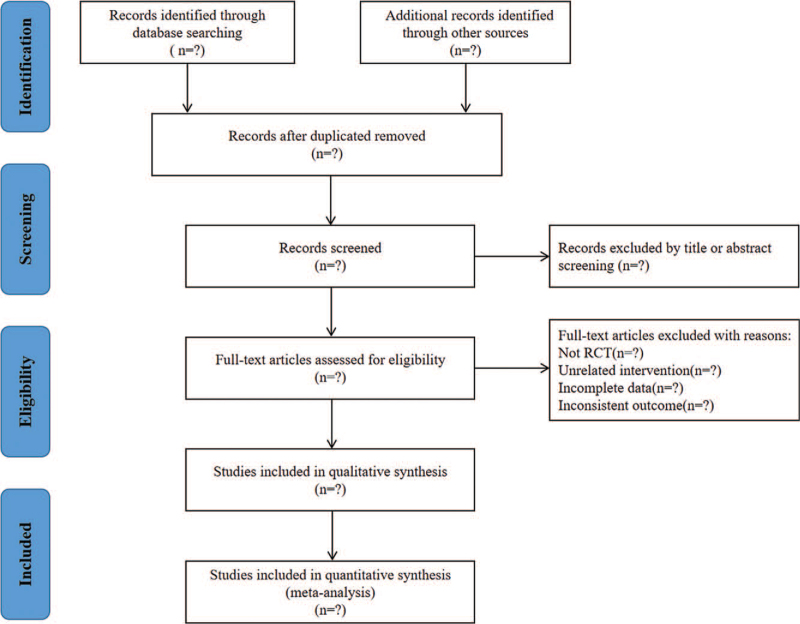
Flowchart of literature screening.

All literatures achieved from electronic databases will be imported into EndNote X9 software for the following screening. Two researchers who are responsible for literature selection must be very familiar with inclusion and exclusion criteria. The screening process is mainly divided into 3 steps. Firstly, duplicated studies will be removed by automatic and manual manner, then irrelevant literatures including animals, reviews, reports will be excluded according to titles and abstracts, finally, we will acquire the eligible studies which are compliant with inclusion criteria through full-text exclusion. The above process of literature selection is presented in Figure 1.

### 2.6 Data extraction and management

After screening the studies, two researchers will independently extract data from the included literatures. The steps of information extraction include designing the table of information extraction, pre-extracting the information, modifying and perfecting the contents of the table, extracting the data by two independent authors and cross-checking the extracted contents at last. Any disagreement will be resolved by discussion until consensus is reached or by consulting a third person. The contents of data extraction are as the following aspects:

Basic information: first author, year of publication, country where the study was conducted, publication type, study type, randomization, sample size, duration, and so on.

Participants: age, gender, diagnostic criteria, inclusion and exclusion criteria, sexual behavior, follow-up time, outcome measures, and so on.

Interventions: intervention methods of treatment group and control group, taking methods, doses, frequency, treatment time, and so on.

Outcomes: CD4^+^, CD8^+^, CD4^+^/CD8^+^, CD45RO, CD45RA, quality of life, Karnovskey Score, opportunistic infections and adverse effects.

### 2.7 Risk of bias assessment

In consideration of all included studies belonged to RCTs, two authors will independently assess the risk of bias of each included study following the domain-based evaluation described in the Cochrane Handbook for Systematic Reviews of Interventions.^[15]^ This assessment tool addresses seven specific items: sequence generation, allocation concealment, blinding of participants and personnel, blinding of outcome assessment, incomplete data, selective outcome reporting, and other issues relating to bias. Each item will be evaluated at three levels: low risk, high risk and unclear.

### 2.8 Data synthesis and analysis

For the extracted data, a quantitative synthesis and analysis will be performed. We will pool the results into Review Manager 5.4 software to conduct a quantitative analysis (meta-analysis) by using a random effects model, with mean difference or standardized mean difference for continuous outcomes and relative risk for dichotomous outcomes, and calculate 95% confidence intervals for each outcome. A 2-tailed *P* < .05 was considered statistically significant.

### 2.9 Heterogeneity analysis

Heterogeneity will be assessed by using the *I* ^*2*^ statistic. We will consider an *I* ^*2*^ value greater than 50% indicative of substantial heterogeneity. When statistical heterogeneity has already been excluded by using a random effects model, if heterogeneity in included studies is still significant, we will choose subgroup analysis or sensitivity analysis to search for clinical and methodological sources.

### 2.10 Subgroup analysis

If significant heterogeneity exists, we will analysis the sources clinically in the following themes:

Treatment time (e.g., < 6 months or > 6 months);

CD4^+^ T cell count baseline values (e.g., < 200/μL or > 200/μL).

### 2.11 Sensitive analysis

To identify the quality and credibility of the results in this review, we will conduct the sensitive analysis, which is aimed at eliminating the possibility of false positives and investigating the reliability of the composite statistical results in our meta-analysis.

### 2.12 Publication bias

We will process the assessment of publication bias by using a funnel diagram though Stata 16 software. When two sides of the funnel plot are not symmetrical, it is suggested that there may be existing publication bias.

### 2.13 Management of missing data

If there is missing or incomplete data in included studies, we will contact original researchers via email for lost information to ensure the comprehensiveness and objectivity of this review. If the missing data are not available, we will only analyze present data or perform some relevant qualitative analysis.

### 2.14 Evidence quality evaluation

The quality of evidence of outcomes will be assessed by using the Grading of Recommendations Assessment, Development, and Evaluation (GRADE) tool. The GRADE system takes the methods of evidence upgrading and downgrading to evaluate the level of evidence of outcomes. The evidence quality will be classified as “high,” “moderate,” “low,” or “very low” according to the GRADE evaluation standards.^[16]^

### 2.15 Ethics and dissemination

Because of all data coming from published studies through electronic databases without directly involving any information of patients, thus there is no ethical approval required. The results of this meta-analysis will be published in a peer-reviewed journal, and make clinicians know more about traditional Chinese herbal medicine combined with HAART in the treatment of HIV/AIDS, in order to formulate the best therapeutic schedule in the future clinical practice for HIV/AIDS patients.

## 3 Discussion

AIDS is a complex disease caused by HIV infection with characteristics of multiple factors, stages and symptoms.^[17]^ The prevention and treatment of HIV/AIDS is still a major public health problem that needs to be solved globally in the 21st century. Although the advent of HAART and its wide application in the world have greatly improved the clinical treatment status of HIV/AIDS, poor immune reconstitution and toxic side effects are still the problems and challenges which are faced in the conventional antiviral therapy.[^18^,^19^] In view of the above limitations of HAART, we should seek for a variety of treatments as adjuvant therapies of HAART to synthetically intervene HIV/AIDS in the post-HAART era.

Since the first case of HIV/AIDS patient was discovered, Chinese clinicians and researchers have been committed to working on the TCM in the treatment of HIV/AIDS. At present, in addition to the routine western medicine treatment, patients with HIV/AIDS in China, especially those who are intolerant of antiviral drugs or failed to antiviral therapy, will be treated with traditional Chinese herbal medicine to improve efficacy and reduce adverse reactions.^[20]^ Although the evidence for the effectiveness and safety of this traditional Chinese and western medicine has been inconclusive, a growing body of studies have shown that this synergistic therapy combing Chinese herbal medicine with HAART has certain advantages in enhancing immunity, improving the quality of life, reducing the occurrence of serious opportunistic infections and alleviating bad effects caused by antivirals.^[21]^

This study aimed to systematically investigate the efficacy and safety of Chinese herbal medicine in cooperation with HAART for HIV/AIDS and provide a reliable reference for the clinical application of Chinese medicine for HIV/AIDS. However, in this systematic review, there may be some limitations mainly occurring in language restrictions, methodological risks and publication bias, which can lead to high heterogeneity and affect the credibility of results. To sum up, we hope the findings of this analysis will provide a helpful evidence for clinicians to develop the best treatment strategy for patients with HIV/AIDS, and also offer scientific clues for researchers to proceed further exploration in this field.

## Author contributions

**Conceptualization:** Zhenzhen Qian.

**Data curation:** Zhenzhen Qian, Yujin Zhang.

**Formal analysis:** Zhenzhen Qian.

**Funding acquisition:** Xiaoli Xie.

**Investigation:** Xiaoli Xie.

**Methodology:** Zhenzhen Qian.

**Software:** Zhenzhen Qian, Xiaoli Xie.

**Supervision:** Junwen Wang.

**Writing – original draft:** Zhenzhen Qian, Yujin Zhang.

**Writing – review & editing:** Zhenzhen Qian, Junwen Wang.
